# The Formin-Homology Protein SmDia Interacts with the Src Kinase SmTK and the GTPase SmRho1 in the Gonads of *Schistosoma mansoni*

**DOI:** 10.1371/journal.pone.0006998

**Published:** 2009-09-10

**Authors:** Thomas Quack, Jürgen Knobloch, Svenja Beckmann, Jérome Vicogne, Colette Dissous, Christoph G. Grevelding

**Affiliations:** 1 Institute for Parasitology, Justus-Liebig-University, Giessen, Germany; 2 Institute for Genetics, Heinrich-Heine-University, Düsseldorf, Germany; 3 UMR8161 – CNRS, Institut de Biologie de Lille, Lille, France; 4 Inserm U547, Université Lille-Nord de France, Institut Pasteur de Lille, Lille, France; University of California Los Angeles, United States of America

## Abstract

**Background:**

Schistosomiasis (bilharzia) is a parasitic disease of worldwide significance affecting human and animals. As schistosome eggs are responsible for pathogenesis, the understanding of processes controlling gonad development might open new perspectives for intervention. The Src-like tyrosine-kinase SmTK3 of *Schistosoma mansoni* is expressed in the gonads, and its pharmacological inhibition reduces mitogenic activity and egg production in paired females *in vitro*. Since Src kinases are important signal transduction proteins it is of interest to unravel the signaling cascades SmTK3 is involved in to understand its cellular role in the gonads.

**Methodology and Results:**

Towards this end we established and screened a yeast two-hybrid (Y2H) cDNA library of adult *S. mansoni* with a bait construct encoding the SH3 (src homology) domain and unique site of SmTK3. Among the binding partners found was a diaphanous homolog (SmDia), which was characterized further. SmDia is a single-copy gene transcribed throughout development with a bias towards male transcription. Its deduced amino acid sequence reveals all diaphanous-characteristic functional domains. Binding studies with truncated SmDia clones identified SmTK3 interaction sites demonstrating that maximal binding efficiency depends on the N-terminal part of the FH1 (formin homology) domain and the inter-domain region of SmDia located upstream of FH1 in combination with the unique site and the SH3 domain of SmTK3, respectively. SmDia also directly interacted with the GTPase SmRho1 of *S. mansoni*. *In situ* hybridization experiments finally demonstrated that SmDia, SmRho1, and SmTK3 are transcribed in the gonads of both genders.

**Conclusion:**

These data provide first evidence for the existence of two cooperating pathways involving Rho and Src that bridge at SmDia probably organizing cytoskeletal events in the reproductive organs of a parasite, and beyond that in gonads of eukaryotes. Furthermore, the FH1 and inter domain region of SmDia have been discovered as binding sites for the SH3 and unique site domains of SmTK3, respectively.

## Introduction

Because of its relevance as one of the most important parasitic infections for humans or animals worldwide, schistosomiasis is an intensely investigated disease [1]–[3]. Blood flukes of the trematode genus *Schistosoma* are the causative agents of schistosomiasis living in the bloodstream of their final hosts. Here they mature and produce eggs that are the principal cause of morbidity, its ensuing pathology may take a fatal course. More than 200 million people are infected and about 700 millions live in endemic regions being at risk [1], [2]. Second in importance only to malaria, schistosomiasis is judged by the World Health Organization as a neglected disease occurring mostly in impoverished urban areas of developing countries. Schistosomiasis is considered not only to be a consequence of poverty, but also a poverty-promoting condition in affected populations [4]. Praziquantel (PZQ) is the only widely applied drug to treat schistosomiasis, but treatment does not prevent reinfection, and first evidence for resistance has been obtained [5]. Therefore, the establishment of alternative strategies to fight schistosomes is desirable. Recently, the great international efforts to find a vaccine have been accelerated by genomic and proteomic studies, but resounding success has not yet been obtained [6]. One approach to identify new candidates for drug design or vaccination is to investigate developmental processes essential for growth or reproduction of this parasite and to identify involved genes/proteins and the networks they are interlinked in.

As the only members of the trematodes, schistosomes have evolved separate sexes. Furthermore, initiation and maintenance of the sexual maturation of the female depend on a continuous pairing contact with the male. Among the consequences of this unique male-female interaction is the differentiation of the reproductive organs, ovary and vitellarium, which are essential for the production of eggs [7]–[10]. Due to their importance for pathology, therapeutical intervention of gonad development by pharmacological blocking of crucial signaling pathways leading to egg production might be an auspicious strategy to combat schistosomiasis.

During the last years compelling evidence has been obtained for signal transduction pathways contributing to gonad differentiation in schistosome females [reviewed in 11], [12]. The best-characterized signaling cascade in *Schistosoma mansoni* is the TGFβ/Smad pathway, of which essential members were identified and characterized [11]. Their interaction was proven, and evidence was obtained for their activity in the female gonads. Also members of other pathways were identified and shown to be expressed in the schistosome gonads. Among these are the Src-kinase homologs SmTK3 and SmTK5 [13], [14]. Studies using specific inhibitors indicated that both Src kinase-containing pathways and TGFβ/Smad pathways contribute to mitogenic activity and egg production [12], [15]. However, in contrast to the characterized members of the TGFβ/Smad pathway, nothing is known about the interaction partners of the Src kinases, nor about the signaling cascades in schistosomes, in which these molecules are involved.

Formin homology (FH) proteins are conserved signal-transduction molecules in eukaryotes. They are involved in actin-mediated processes controlling cell and tissue architecture playing important roles in cell polarity, cell-cell interactions, gastrulation, or cytokinesis [16], [17]. Once thought to be only molecular scaffolds that indirectly affect cellular functions through the binding of other proteins, *in vitro* studies have shown their role as actin nucleators in the formation of new filaments. FH proteins are defined by the presence of FH1 and FH2 regions of homology to the mouse limb deformity proteins, and many family members contain additional conserved motifs. The prolin-rich FH1 region interacts with potential effector proteins including the actin-binding protein profilin, WW domain- containing proteins, or SH3 domain-containing proteins such as Src kinases. The FH2 domain functions in actin binding and cytoskeletal reorganization. The armadillo repeat/dimerization region (ARR/DIM), previously called FH3 domain, appears to be involved in dimerization and subcellular localization [16]–[18].

A subgroup of the formin family consists of the diaphanous-related formins (Drf or Dia) [17], [18]. They are characterized by two additional conserved domains: an N-terminal Rho GTPase binding-domain (RBD or GBD), and a C-terminal diaphanous autoregulatory domain (DAD). The RBD domain is a target of activated Rho proteins, small GTPases cycling between an active GTP-bound and an inactive GDP-bound conformation [19]. Dia proteins can act as effectors for Rho-GTP. Rho-family proteins on their part are involved in the regulation of the actin cytoskeleton, but also in membrane trafficking, transcriptional control, regulation of cell adhesion, and cell cycle progression [19], [20]. Direct interactions of RhoA with mDia1 and mDia2 have been demonstrated in mouse cells as well as Src kinase/Dia interaction, indicating Rho-Dia-Src cooperative activity in the regulation of actin dynamics [21]. In schistosomes, the Rho-GTPases SmRho1 was identified, which exhibits 71–75% identity and about 85% similarity to human Rho A, B, and C proteins [22], [23]. Furthermore, SmRho1 was able to complement a *Saccharomyces cerevisiae* Rho1 null mutation [23].

Members of the Src family of cellular protein tyrosine-kinases (PTKs) interact among others with growth factor receptors. PTK pathways involving Src have been shown to cross-talk with Rho pathways during intracellular signaling processes [21], [24]. Besides a highly conserved tyrosine kinase domain with catalytic function, Src kinases contain three highly conserved Src-homology (SH) domains. Among these, the SH2 and SH3 domains are responsible for the binding of interaction partners within signaling cascades [25]. SH2 domains bind to short amino acid sequences of potential upstream binding partners containing a tyrosine, whose phosphorylation enhances binding affinity. The SH3 domain contacts proline-rich sequences (PxxP) of potential downstream partners, but also atypical sequence motifs [25], [26]. The SH4 domain, finally, represents an amino-terminal myristoylation site, required for membrane association [25], [27]. Between the SH4 and SH3 domains of Src kinases is a poorly conserved unique region (called unique site or unique domain), which is of unclear function and characteristic for each Src-family member. In the past, weak experimental support was found for a role of the unique site to support the binding efficiency to interaction partners [27]. More recently, first evidence was obtained for an independent role of the unique site as a protein-protein interaction region. The unique site of marine n-Src was found to bind to the oxidoreductase domain of the human NADH dehydrogenase subunit 2 [28].

One aim of our study was to identify downstream binding partners of the Src kinase SmTK3. Using a newly established Y2H cDNA library of mixed-sex adult *S. mansoni*, and a bait construct of SmTK3 (SH3-domain and unique site) as probe we isolated among other potential interaction partners the diaphanous homolog SmDia. Besides genomic and transcriptional profiling we demonstrate that SmTK3/SmDia binding is mediated by the SH3 domain as well as the unique site of SmTK3 and the inter-domain region as well as the N-terminal part of the FH1 domain of SmDia. Thus first evidence is provided for a diaphanous target region of the unique site of Src kinases. Furthermore, we discovered the Rho-GTPase homolog SmRho1 of *S. mansoni* as a first pivotal interaction partner of SmDia. *In situ* hybridization data confirmed colocalization of all three molecules in the reproductive organs of both genders. In conclusion we suggest the existence of a minimum of two conserved cooperative signal-transduction pathways, involving Rho and Src, that bridge at Dia probably organizing cytoskeletal events in the gonads of adult *S. mansoni*.

## Materials and Methods

### Parasite stock

A Liberian isolate of *Schistosoma mansoni* [29] was maintained in *Biomphalaria glabrata* as intermediate host and in Syrian hamsters (*Mesocricetus auratus*) as final host. Adult worms were obtained by hepatoportal perfusion 42 days post infection and gently separated using a fine brush.

### Isolation of nucleic acids

Isolation of genomic DNA and total RNA from larval and adult schistosomes was done as described elsewhere [15], [29]. Extraction and purification of poly(A^+^)-RNA from schistosomes for yeast two-hybrid library-construction was done using a guanidinium thiocyanate-based isolation method and caesium chloride centrifugation as described by Sambrook et al. [30].

### Generation of a Schistosoma mansoni yeast two-hybrid library

The Matchmaker III system (Clontech) was used to generate a *S. mansoni* Gal4-based yeast two-hybrid (Y2H) library. For experimental details, see the appropriate user manual (Matchmaker library construction & screening kit user manual, Clontech). Briefly, poly(A^+^)-RNA was obtained by a guanidinium-thiocyanate extraction procedure and CsCl centrifugation [30]. First-strand cDNA synthesis was done with 1.2 µg poly(A^+^)-RNA from adult, mature male and female worms and oligo(dT)-priming. After tailing reaction and amplification of double stranded cDNA by long-distance PCR, fragments up to 500 bp were discarded by chromatography-based size selection. Finally, cDNA was cloned by a recombination step in frame with the Gal4 activation domain (Gal4 AD) into pGADT7-Rec (prey vector; leucin selection marker LEU; Clontech). Transformants were selected on synthetic dropout (SD) medium lacking leucine, pooled, and aliquots stored at −80°C.

For subsequent library screening, the mating protocol was followed (Clontech). For this, two yeast strains were used, the library-containing strain AH109 (Mat a; containing the reporter genes ADE2, HIS3, and lacZ) and the bait-containing strain Y187 (Mat α; ADE2, lacZ), whose reporter genes were under the control of Gal4 upstream activating sequences (UASs) and TATA boxes. As bait vectors for library screening, the shuttle plasmids pGBKT7 or pGBKT9 (Clontech) were used, which both express proteins fused to the Gal4 DNA-binding domain (DNA-BD). These vectors carry the tryptophan nutritional marker TRP1 for selection in yeast. In addition, the prey vector pACT2 was used, which contains the Gal4 AD and the LEU selection marker.

### Cloning procedures

**Figure 1:**
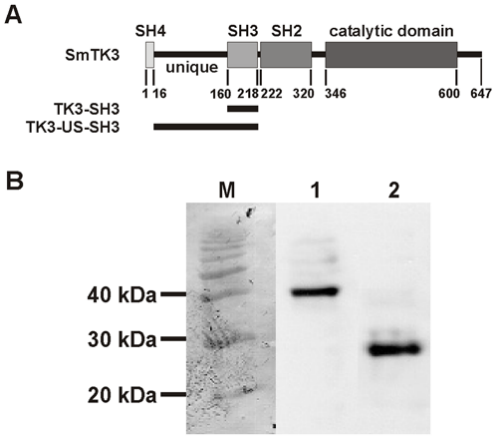
Schematic structure of SmTK3 and expression of the SmTK3-bait proteins in yeast. A: The diagram shows the structure of SmTK3 with the Src-homology domains 2–4 (SH2-SH4), the tyrosine-kinase domain (catalytic domain), and the unique site (unique). The parts of SmTK3 used as baits for Y2H-library screening are shown below the diagram (TK3-SH3 and TK3-US-SH3). B: Western blot analyses of protein lysates of SmTK3-SH3(+/-US) pGBKT9-transformed Y187 yeast-cells to confirm the expression of the bait constructs. Using an anti-Gal4 antibody, bands of the expected sizes for the fusion proteins Gal4 DNA-BD/TK3-US-SH3 (lane 1) and Gal4-DNA-BD/TK3-SH3 (lane 2) were detected. Marker (M): 10 kDa ladder (Gibco BRL).

Cloning candidate genes or gene fragments into vectors for Y2H analyses was done as follows. One bait vector containing the 5′ unique site combined with the SH3 domain (611 bp fragment) of SmTK3 was constructed to screen for “downstream” interaction partners [13]. Another bait vector was cloned, which contained the single 185 bp long SH3 domain amplified by the primer combination TK3-SH3-1 (5′-GGAGAATTCGGGCAGTTTGTTGCTTTAC-3′; containing an *Eco*RI restriction site) and TK3-SH3-2 (5′-TTCGTCGACGGATTCCAAACTGGTAAC-3′; containing a *Sal*I restriction site). This vector was used for subsequent comparative Y2H analyses to investigate the role of the unique site for the binding efficiency of potential interaction partners (Fig. 1A). Each bait fragment was cloned in frame with the Gal4 DNA-BD into pGBKT9.

As a bait construct to analyze SmDia/SmRho1 interaction, the Rho binding domain (RBD) of the schistosome gene SmDia was subcloned. To this end, the SmDia-specific primer pair RBD-5′ (5′-CGGGATCCCTAGAAGTAGCGAGTCACCA-3′; containing a *Bam*HI restriction site) and RBD-3′ (5′-CCAATGCATTGGTTCTGCAGGCCTTTAGCATTTACAAAGAGC-3′; containing a *Pst*I restriction site) was used for PCR. Amplification products of the expected size were eluted from an agarose gel and cloned via *Bam*HI/*Pst*I into the vector pGBKT7. After cloning, the insert was sequenced to confirm the identity and integrity of the open reading frame of the fusion of the Gal4 DNA-BD and RBD domain within pGBKT7-DiaRBD.

To avoid unspecific membrane targeting by prenylation of the C-terminally located CAAX motif, SmRho sequence variants without CAAX were generated by a PCR-based strategy using the primer pair Rho-5′Bam (5′-CGGGATCCTAATGGCGAGTGCGGTACG-3′; containing a *Bam*HI restriction site) and Rho-3′Bam (5′-CGGGATCCCTAGAAGTAGCGAGTCACCA-3′; containing a *Bam*HI restriction site). Amplification products of the expected size were eluted from an agarose gel and cloned via *Bam*HI restriction into pACT2. Sequencing confirmed the integrity of the open reading frame of the fusion protein insert within pACT2-Rho1. Two additional sets of primer pairs were designed to induce mutations by site-directed mutagenesis into this sequence by a whole-plasmid (circular template) PCR approach (SmRhoG15V s: 5′-GTTGGAGATGTTGCATGCGG-3′/SmRhoG15V as: 5′-CCGCATGCAACATCTCCAAC-3′; both primers overlap to complementary introduce a single point mutation leading to the amino acid exchange G15V; SmRhoQ64L s: 5′-CTGCTGGCCTAGAAGATTATG-3′/SmRhoQ64L as: 5′-CATAATCTTCTAGGCCAGCAG-3′; both primers overlap to complementary introduce a single point mutation leading to the amino acid exchange Q64L). PCR amplifications were done in 50 µl total volume using standard conditions (further technical details are available upon request). Amplification products were *Dpn*I-digested and 1 µl used *E. coli* transformation (NovaBlue GigaSingles™; Novagen). Candidate clones were sequenced to confirm the successful introduction of the point mutations.

For comparative β-Gal liquid assays deletion constructs of the SmDia inter-domain region have been generated (ID1 and ID2) by an *in vitro* mutagenesis approach employing the reverse complementary primer pairs SmDia#402 (sense: 5′-CAGAGTGGCTATTATGGCCGGGGAATGATGGTGTCGATCCTGATCC-3′ antisense: 5′-GGATCAGGATCGACACCATCATTCCCCGGCCATAATAGCCACTCTG-3′) and SmDia#432 (sense: 5′-CAGAGTGGCTATTATGGCCGGGGGCTGACGCTTCAACTCGTGTTGAG-3′antisense: 5′-CTCAACACGAGTTGAAGCGTCAGCCCCCGGCCATAATAGCCACTCTG-3′), respectively. The underlined part of the primer sequences correspond to the C-terminal end of the Gal4-AD, whereas the dotted and dashed parts of the primer sequences represent SmDia starting at amino acid position 402 or 432, respectively. PCR amplifications were done in a total volume of 50 µl containing 25 ng DNA (pGADT7-SmDia-S141), 0.5 µM of each primer, 200 µM of each deoxynucleotide (dATP, dTTP, dCTP, dGTP), and 3 U *pfu* polymerase (Promega). The temperature cycling profile was as follows. After an initial denaturation step at 95°C for 30 sec, cycling was done at 95°C, 30 sec/55°C, 1 min/68°C, 20 min for 18 cycles. Amplification products were digested with 20 U *Dpn*I for 1 hour at 37°C and 1 µl was subsequently used for *E. coli* transformation (10-β High Efficiency Competent®; NEB). Candidate clones were screened by colony PCR and sequenced to confirm the deletion of 111 bp (ID1) or 201 bp (ID2).

The 5′ region of the diaphanous gene was identified by a genomic approach. To this end the “Universal GenomeWalker” kit (BD Bioscience) was used. For the construction of 4 sublibraries 5 µg purified DNA of *S. mansoni* adults was digested each with *Dra*I, *Eco*RV, *Pvu*II and *Ssp*I. Ligation of anchor sequences was done according to the instructions of the manufacturer. Nested-PCR amplification of diaphanous 5′-fragments was done with primers directed against the anchor sequence (AP1 flanking: 5′-GTAATACGACTCACTATAGGGC-3′; AP2 nested: 5′-ACTATAGGGCACGCGTGGT-3′), and two diaphanous cDNA sequence-derived primers, one flanking primer (GWP3: 5′-CCAGGGCTTTAACTAAAGGCATGAAGC-3′), and one nested primer (GWN3: 5′-CTGGTGTCCCATTAAGTTCCGCACTAA-3′). PCR amplifications were done in a total volume of 50 µl containing 100 ng DNA, 200 µM of each primer, 250 µM of each deoxynucleotide (dATP, dTTP, dCTP, dGTP), and 5 U *Taq* polymerase (TripleMaster, Eppendorf). According to the TripleMaster protocol, a two-step temperature cycling profile was performed (Eppendorf). After an initial denaturation step at 94°C for 2 min, cycling was done at 94°C, 25 sec and 72°C, 3 min for initial 7 cycles, followed by 94°C, 25 sec and 70°C, 3 min for 32 cycles. A final elongation step was done for 7 min at 67°C. Amplification products were identified by agarose gel electrophoresis, eluted from the gel, and cloned into pDrive (Qiagen). Sequencing was done commercially (Agowa).

### Screening of the yeast two-hybrid library

The SmTK3 bait construct (unique site and SH3 domain in pGBKT9) was transformed into yeast strain Y187 by the lithium acetate method (Yeast Protocols Handbook, Clontech). For library screening, bait-expressing Y187 cells were mated with library-containing AH109 cells. A mating efficiency of 7.6% was obtained, a value which substantially exceeded the required minimum of 2% (Clontech). The selection for diploid yeast cells expressing interacting proteins was carried out on synthetic dropout medium lacking the amino acids tryptophan, leucine, histidine and adenine (Trp^−^/Leu^−^/His^−^/Ade^−^). Approximately 2.5×10^6^ clones were screened. However, under statistic consideration, only about one third of the clones expressed the recombinant insert DNA leading to an effectively screened number of approximately 8.3×10^5^ clones. For selection purposes, β-Galactosidase (β-Gal) filter assays were performed using X-Gal as substrate according to the manufacturer's instructions (Clontech). Plasmid DNA from positive yeast clones was isolated as previously described [31], and then transformed in HB101 competent bacterial cells. By this procedure, cDNA-containing pGADT7 prey plasmids were separated from bait plasmids, recovered, and commercially sequenced. For further analyses, candidate prey clones were co-transformed with bait plasmids into yeast cells. To confirm specific protein-protein interactions, the two selective procedures were repeated and, additionally, liquid β-Gal assays were done for quantification using ONPG as substrate. For experimental details see the Matchmaker library construction & screening kit user-manual and the Yeast Protocols Handbook (Clontech).

### Isolation and characterization of yeast proteins

Total protein extracts from yeast cells were obtained as described in the Yeast Protocols Handbook (Clontech) and analyzed by standard SDS PAGE and blotting [32].

The expression of the bait fusion-proteins was proven by standard Western blot analyses (Fig. 1B) using an anti-Gal4 primary mouse antibody (1∶500; Santa Cruz Biotechnology) and an alkaline phosphatase-coupled secondary goat anti-mouse antibody (1∶10.000; Sigma). Detection was performed by chemiluminescence using CDP star as substrate (Amersham).

### Northern blotting

For Northern blotting, 10 µg total RNA were size-fractionated by 1.5% denaturing agarose gel electrophoresis. As probes, parts of SmDia gene such as the S4 fragment, or the S3 fragment were used. The latter was amplified by PCR using the primer combination SIII5′ (5′-ATGGGAGGTATTCCGCCAC-3′) and SIII3′ (5′-AGGGCTTTAACCAGCTGCTC-3′) before labeling. Blotting and hybridization were done under stringent conditions, as described for Southern blots.

### RT-PCR and quantification

RT-PCRs (reverse transcriptase PCRs) and qPCRs (quantitative PCRs) were done stepwise in separate reactions. *S. mansoni* total RNA was isolated from adult and larval stages and converted to cDNA using Superscript II (Invitrogen) according to the instructions of the manufacturer. About 0.5 to 1 µg total RNA were used, and the synthesis of SmDia cDNA first-strands was initiated with 1 µM oligo-(dT) or a gene-specific primer (SIV3′: 5′-TCCTTTAGTGCATCTACC-3′). One tenth of the reverse transcription reaction was used for standard PCR amplifications in 25 µl with different primers (g5′: 5′-ATTGCGTTCCGTTACAGGCA-3′; or SIV5′, see above).

For semiquantitative RT-PCR, conditions for cycle numbers and linear amplification were determined using the house keeping gene protein disulfide isomerase [PDI; 33] as the standard reference. The determination of signal intensities resulting from the amplification reactions was done by densitometric quantification. The relative values were obtained by integration using the ScionImage software (Scion Image for Windows – version Beta 4.0.2). Different exposures of each experiment were scanned with a ScanJet 4c (Hewlett Packard) and taken for the evaluation. Results were summarized and presented as graphics using Microsoft Excel.

For quantitative PCR cDNAs from male and female worms were used as templates for amplification using the SYBR® Green PCR Master Mix and the ABI PRISM 7000 sequence detection system (Applied Biosystems). Primers specific for SmDia (Fwd 5′- TTTAGCACAAAGCCAGCTAAGGTGA-3′ and Rev 5′-GCCATCTGATGTAGAATCAGACACCAT-3′) and SmPDI (Fwd 5′- GTCAAACTGTATGCTCCTTGGTGTG-3′ and Rev 5′-AACTGGAGCAAGAGCCTTGCA-3′) were designed by the Primer Express Program (Applied Biosystems) and used for amplification in triplicate assays. For graphical representation of quantitative PCR data, raw cycle threshold (Ct values) obtained for female worms were deducted from the Ct value obtained for male worm transcripts using the delta-delta Ct (ΔΔCt) method [34], with DPI gene levels serving as the internal standard.

### *In situ* hybridization

Adult worm pairs were fixed in Bouin's solution (picric acid/acetic acid/formaldehyde as ratio 15∶1∶5) as described elsewhere [13] and embedded in paraplast (Histowax, Reichert-Jung). Sections (5 µm) were incubated in xylol to remove the paraplast. After re-hydration, proteins were removed by proteinase K treatment (final concentration 1 µg/ml), and the sections were dehydrated. For hybridization, *in vitro* transcripts were labeled with digoxigenin according to the protocol of the manufacturer (Roche). Labeled sense and antisense transcripts of SmDia or SmRho1 subclones were size-controlled by gel electrophoresis. To prove their quality, transcripts were blotted and detected with alkaline phosphatase-conjugated anti-digoxigenin antibodies, naphthol-AS-phosphate, and Fast Red TR (Sigma). The *in situ* hybridization was done for 16 h at 52°C. Sections were stringently washed up to 0.1×SSC, and detection was achieved as described for transcripts.

### *In silico* analyses

The following public domain tools were used: Swiss-Prot (http://www.expasy.ch/sprot/sprot-top.html), ClustalW (http://www.clustalw.genome.ad.jp/), BLAST (http://www.ncbi.nlm.nih. gov/BLAST/), PROSITE (http://www.expasy.ch/prosite/), InterPro (http://www.ebi.ac.uk), Motif (http://motif.genome.ad.jp), Fasta (http://www2.ebi.ac.uk/fasta3/).

For comparison of clone sequences obtained in this study the Welcome Trust Sanger Institute *S. mansoni* OmniBlast server was used (http://www.sanger.ac.uk/cgi-bin/blast/submitblast/s_mansoni/omni).

## Results

### Screening a *S. mansoni* yeast two-hybrid library for interaction partners of SmTK3

To identify interaction partners of SmTK3, which could help to unravel signalling cascades SmTK3 is involved in, a Y2H cDNA-library was constructed with poly(A^+^)-RNA from adult male and female *S. mansoni*. This library comprised about 9×10^8^ independent clones and should represent the whole adult schistosome transcriptome since the estimated number of genes in the *S. mansoni* genome adds up to <2×10^5^ [35]. The average insert size of the library was determined by restriction analyses of 30 randomly selected clones and was found to be 1.2 kb with a maximum of >3 kb (result not shown). Clones without insert or with inserts shorter than 500 bp were not detected.

For library screening to identify molecules acting “downstream” of SmTK3 in a signaling hierarchy, a bait construct was cloned containing the unique site and the SH3 domain (TK3-US-SH3) of SmTK3 (Fig. 1A). It is known that SH3 domains bind to proline-rich regions of specific downstream binding partners of cellular kinases [36], which is supported by the unique site [27], [28]. The expression of this bait construct and a second one, which contains only the SH3 domain (TK3-SH3) serving subsequent binding studies (see below), was confirmed by Western blot analyses (Fig. 1B). Screening of the *Schistosoma* library was performed by using the yeast-mating technique. Out of 123 initial prey clones obtained, 39 clones remained positive following pre-selection on selective growth media and β-Gal filter assays. According to analytical restriction digests using *Dra*I, these clones were arranged in 9 groups (A–I, Table 1). Sequencing of all clones confirmed this grouping, and *in silico* analyses (using templates≥550 bp) revealed for 6 groups sufficient evidence for identity (E-values 2e-136 - 3e-18). The clones of these groups represented diaphanous (group A), an eukaryotic translation initiation factor (eIF4γ2b) (group B), the BAF60 subunit of the SWI/SNF complex (group C), a YME1-like metalloprotease (group D), a mRNA (guanine-7) methyl transferase (group E), and a quinolinate phosphoribosyltransferase (Qprt) (group F). For 2 groups weak homologies (1e-02, 2e-03) were found to an SH3 binding protein from *Rattus norvegicus* (group G), and a Smad protein (Hrssmad 2/3) from *Halocynthia roretzi* (group H). No significant homology (2.7) was detected for 1 group (group I), which pooled most clones (E18). For the clones of this group very weak similarity was detected to pericentrin B from *Homo sapiens* (Table 1).

**Table 1:** Identified downstream binding partners of SmTK3.

Clone group	homology	Accession number	e-value	clone	insert size (bp)
**A**	diaphanous homolog 3	NM_019670	2e-136	S439	2,719*
	(Diap3)			S340	2,600*
	[*Mus musculus*]			S283	2,425*
				S141	2,400*
				S428	2,100*
				S010	2,000*
				S433	1,950*
				S440	1,300*
**B**	eukaryotic translation initiation factor	BC127398	9e-35	S037	2,800
	(eIF4γ2b)			S009	2,700
	[*Danio rerio*]			S021	2,400
				S129	1,400
				S279	1,400
				S096	900
**C**	SWI/SNF complex 60 KDa subunit	AAC52794	1e-127	S052	1,400
	(BAF 60)			S138	1,400
	[*Mus musculus*]				
**D**	YME1-Like	NM_066897	3e-122	S139	1,200
	(Yeast Mitochondrial Escape)				
	AAA protease family member (ymel-1)				
	[*Caenorhabditis elegans*]				
**E**	RNA (guanine-7-) methyltransferase	NM_001098942	3e-59	S039	1,150
	(RNMT)				
	[*Bos taurus*]				
**F**	quinolinate phosphoribosyltransferase	NM_133686	3e-18	S083	1,700
	(Qprt)				
	[*Mus musculus*]				
**G**	SH3-domain binding protein	RNU31159	1e-02	S085	2,000
	(CR16)				
	[*Rattus norvegicus*]				
**H**	Hrsmad2/3	AB058904	2e-03	S301	1,350
	[*Halocynthia roretzi*]				
**I**	pericentrin B	AF515282	2.7	S011	1,700
	(PCNT2)			S017	1,400
	[*Homo sapiens*]			S023	1,100
				S026	1,100
				S030	1,050
				S047	1,300
				S076	1,200
				S090	1,200
				S108	1,400
				S114	1,200
				S281	1,100
				S288	1,100
				S308	1,200
				S329	1,200
				S445	1,100
				S448	1,100
				S455	1,100
				S456	1,100

Clones identified by Y2H analyses using the SmTK3 baits TK3-SH3 or TK3-US-SH3. The appropriate clone numbers are given as well as their insert sizes (bp), and the levels of homology of their sequences (E-values). * = fully sequenced clones.

* completely sequenced clones.

### Qualitative and quantitative analyses of interaction partners

**Figure 2:**
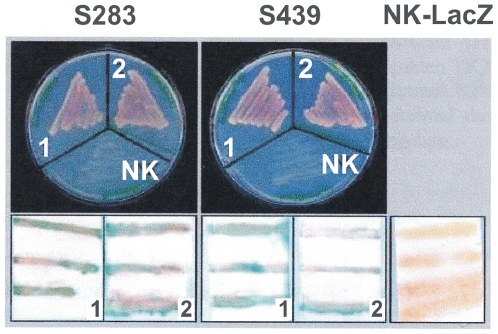
SmTK3/SmDia-interaction in yeast. To verify interactions prey clones were retransformed together with the baits TK3-SH3 (1) or TK3-US-SH3 (2) in yeast AH109 cells and grown under selective conditions (Trp^−^/Leu^−^/His^−^/Ade^−^). As a representative example two clones of the diaphanous related formin (DRF) SmDia (S283 and S439) are shown (top). β-Gal filter assays also confirmed interaction in each case (blue color; bottom). No growth (NK on the agar plates) or β-Gal activity (NK-LacZ) were observed when yeast cells were transformed exclusively with the bait TK3-US-SH3, or co-transformed with the mentioned representative clones and lamin C (negative control, result not shown).

To exclude artificial interactions, a representative clone of each group was retransformed in yeast AH109 cells together with the bait constructs TK3-US-SH3 or TK3-SH3. In each case cells grew under selective conditions on synthetic dropout agar plates (Trp^−^/Leu^−^/His^−^/Ade^−^). β-Gal filter-assays also confirmed interaction in each case (Fig. 2, representative example). No growth or β-Gal activity were observed when yeast cells were transformed exclusively with the bait construct TK3-US-SH3, or co-transformed with the mentioned representative clones and lamin C, an unspecific prey control (Fig. 2; result not shown). These tests demonstrated the credibility of the observed interactions of each clone group.

**Figure 3:**
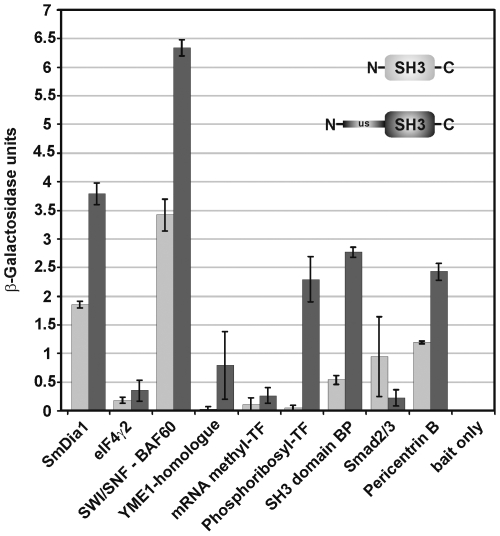
Comparative liquid β-Gal assays to determine the relative binding strengths of potential SmTK3 interaction partners. Comparative β-Gal liquid assays were performed to determine the relative binding strength of the identified interaction partners (n = 6). A representative member of each clone group was re-transformed in yeast cells (AH109) together with TK3-SH3 (light grey) or TK3-US-SH3 (dark grey). The tested clones were (from left to right): diaphanous (SmDia), the eukaryotic translation initiation factor (eIF4γ2b), the BAF60 subunit of the SWI/SNF complex (SWI/SNF-BAF60), the YME1-like metalloprotease (YME1-homologue), the mRNA (guanine-7) methyl transferase (RNA methyl TF), the quinolinate phosphoribosyl-transferase (phosphoribosyl TF), the SH3 binding protein (SH3 domain BP), the Smad protein (Smad2/3), and pericentrin B. As negative control, yeast cells were transformed with TK3-US-SH3 only (bait only).

To investigate the relative binding strengths of the potential interaction partners, representative members of each clone group were re-transformed in yeast cells together with TK3-SH3 or TK3-US-SH3 for comparative liquid β-Gal assays (Fig. 3). This quantitative test again confirmed interaction, and, in addition, demonstrated the strongest binding of TK3-SH3 with BAF60 and diaphanous. In both cases, binding was enhanced in the presence of the unique site. This applied also to the representatives of the other clone groups except Smad 2/3 (group H), where the SH3 domain alone led to a stronger interaction. In two cases, the YME1-like metalloprotease (group D) and Qprt (group F), the unique site seemed to be exclusively responsible for the binding activity. As expected, no β-Gal activity was measured when control yeast cells were transformed with the bait plasmids only. These findings correspond to previous reports suggesting that the unique site may reinforce binding of TKs to their substrates [27], or may even act itself as a binding domain [28].

### Diaphanous (SmDia) cloning and sequence analyses

Since the most frequently occurring clones within a clearly allocable group represented diaphanous homologs we started to characterise these first. The inserts of the 8 diaphanous clones varied in sizes from 1300 to 2719 bp, all of these were completely sequenced (Table 1, group A). Multiple alignment data-base analyses comparing the *S. mansoni* diaphanous cDNAs with diaphanous homologs of vertebrates and invertebrates indicated that none of the cloned schistosome sequences contained a complete 5′-cDNA region (result not shown). The 5′-part of the armadillo repeat region (ARR) was missing as well as the Rho-binding domain (RBD), both of which are characteristic for diaphanous proteins [18].

Since 5′-RACE experiments and cDNA library screenings failed to deliver the missing diaphanous sequences, we decided to perform a genome walking approach. Genomic DNA libraries of *S. mansoni* were made and screened by a PCR approach using nested-primers against defined anchor sequences and the known diaphanous 5′-cDNA end. Amplification products of about 900 bp were obtained and cloned. Sequence analyses showed that the clones contained the known diaphanous 5′-cDNA end and further parts including the complete ARR and RBD regions. Also a methionine was identified that could represent the translational start site, indicating that the complete 5′-part was identified. To confirm that the cloned genomic DNA sequence was part of the transcribed region without an intron, RT-PCR experiments were done. With the primer pair g5′and SIV3′ an amplification product of the expected size of about 1.3 kb was obtained, whereas a control RT-PCR without reverse transcriptase reaction did not result in any product (results not shown). Sequence analysis of the cloned RT-PCR product showed a perfect correspondence to the sequence cloned from the genomic library indicating that there is no intron in this region of the gene, which we finally called SmDia.

**Figure 4:**
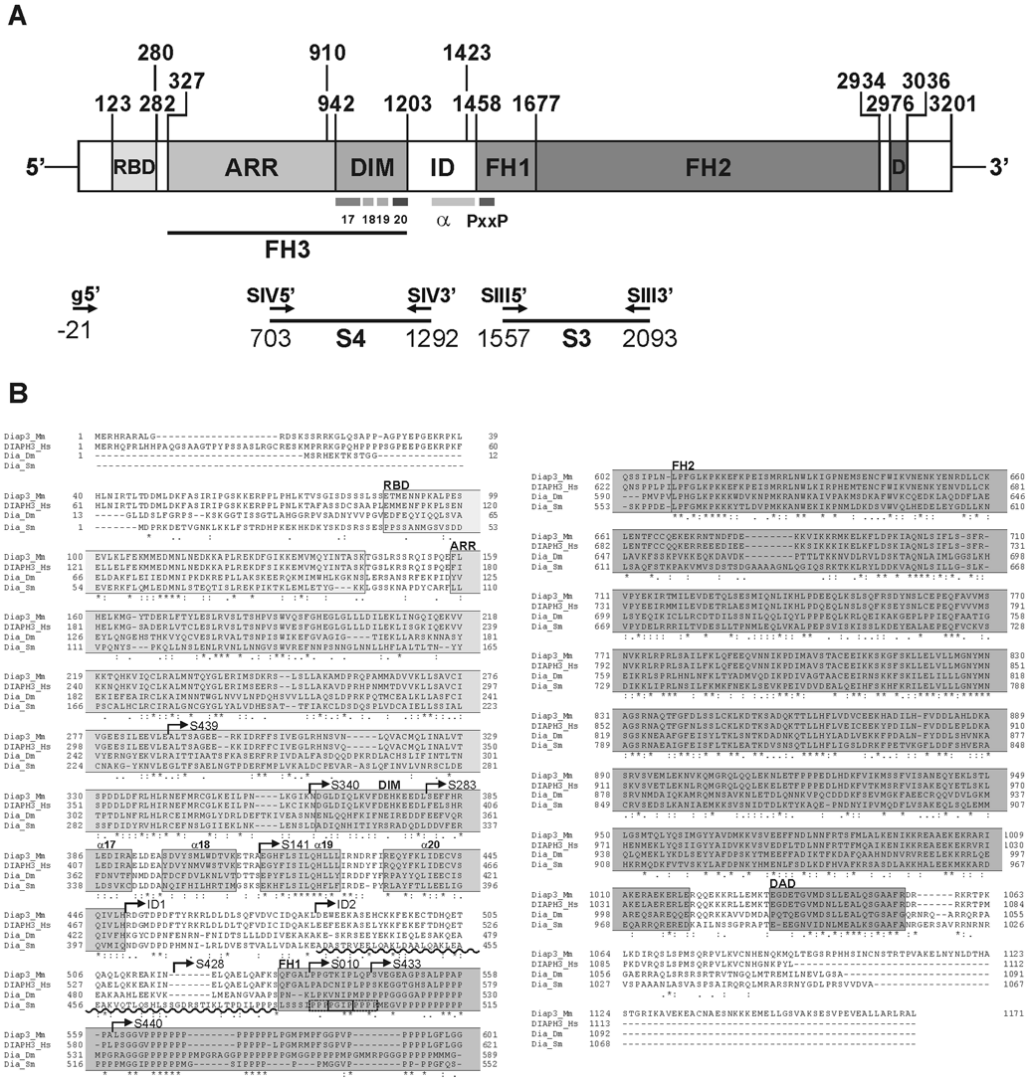
Schematic structure of SmDia and multi-alignment of different Dia homologs. A: Diagram of the structure of SmDia, which consists of an N-terminal Rho GTPase binding-domain (RBD), an armadillo repeat/dimerization region (ARR/DIM; formerly called FH3 domain), the inter-domain (ID) region, the forming-homology domains 1–2 (FH-domains 1–2), and a diaphanous autoregulatory domain (D) at the C-terminus. Above the diagram, nucleotide positions are indicated. Within the DIM region there are 4 α-helices (17–20). The predicted α-helix (α) within the ID region is depicted. At the 5′-part of the FH1 domain a PxxP motif occurs, which is necessary for binding to the SH3 domain of Src kinases. Below the diagram, PCR primers and their positions are indicated (g5′, SIV5′, SIV3′, SIII5′, SIII3′) and the probes (S4, S3) used for Southern-, or Northern-blot analyses (see text for details). B: Multialignment of the SmDia amino acid sequence (Dia_Sm; FN179407) with diaphanous sequences from mouse (Diap3_Mm; NP_062644), human (DIAPH3_Hs; AAW78862), and *Drosophila* (Dia_Dm; AAA67715). Boxed grey areas indicate domains characteristic for diaphanous proteins such as the terminal Rho GTPase binding-domain (RBD), armadillo repeat region (ARR), the dimerization region (DIM), the forming-homology domains 1 (FH1) and 2 (FH2), and the diaphanous autoregulatory domain (DAD). In addition, the 4 α-helices within the DIM domain (α17–α20), the predicted α-helix within the ID region (sinuous line). Furthermore, the start points of the partial, N-terminally shortened clones (S439–S440) identified during the Y2H screening as well as the deletions constructs generated by *in vitro* mutagenesis (ID1 and ID2) are shown. These clones were used for SmDia/SmTK3 binding-studies (Fig. 7). The small box within the FH1 domain of SmDia (Dia_Sm) indicates the putative PxxP-motif region.

Sequence analyses revealed a length of the SmDia coding region of 3201 bp yielding a protein of 1067 amino acids. The 5′-UTR comprised 116 bp and included an in-frame stop codon. The 5′-UTR contained a putative TATA-box (TATAAG) at position −59 to −54 with respect to the ATG codon, but no perfect Kozak consensus sequence for the transcriptional start site [37] was found. The 3′-UTR has a length of 109 bases between the TGA stop codon and the polyA-tail, and contains two identical putative polyadenylation sites (CATAAA at positions 3281–3286 and 3294–3299), the latter one preceding the polyA-tail by 14 bases. A Blast search through the schistosome genome assembly [38; version 3.1] identified a single contig and a single scaffold containing the complete SmDia gene sequence (Smp_contig 0192386940; Smp_scaff 000227), confirming that SmDia has no introns. This had been indicated by the results mentioned above, but also by further PCR experiments using genomic DNA and primer combinations that covered the whole sequence. In each case, amplification products were obtained, whose size corresponded to the appropriate cDNA fragments (results not shown). Since the *Schistosoma* genome project is still not completely finished, Southern-blot analyses were performed which finally confirmed the single-copy gene status of SmDia (result not shown). With the exception of 7 nucleotide differences, this contig sequence is identical to the cDNA sequence identified in our study (accession number FN179407). The detected sequence variations are likely due to strain-specific and/or allelic differences resulting in three silent mutations, the deletion of one proline within a proline-rich region, and one amino acid substitution (glycine/arginine). These minimal discrepancies most likely have no significant influence on protein function since a strech of 15 prolines is shortened by only one, and the glycine/arginine-exchange occured within the non-conserved C-terminal region downstream of the autoregulatory domain (DAD). BLASTX analyses of the SmDia full-length sequence revealed that the deduced amino acid sequence has homology to the family of diaphanous-related formins. Figure 4 shows a diagram of the structure of the molecule (A) and a multi-alignment (B) with proteins revealing the highest degree of homology to SmDia (48–56% identity, 65–71% similarity). As expected for members of this protein group, SmDia contains the characteristic domains for Rho binding (RBD), dimerization (DIM), two formin-homology regions (FH1, FH2), and autoregulation (DAD, Fig. 4B).

### Transcription analyses of SmDia

**Figure 5:**
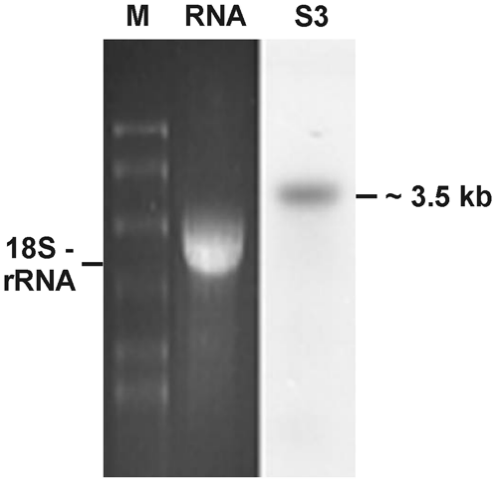
Northern-blot analyses of SmDia transcripts. Northern-blot analyses with RNA (left side) of adult schistosomes (mixed-sex). As probe for the filter, a radioactively labeled part of SmDia (fragments S3, see Fig. 4A) was used (right side). Sizes (kb) are indicated. M = RNA molecular weight marker.

RNA of adult worms (mixed sex) was used to investigate the transcription of SmDia by Northern Blot analyses. As probes, different, radioactively labeled parts of SmDia (fragments S3 and S4, see Fig. 4A) were used. In each case, a distinct signal of approximately 3.5 kb was observed with both total RNA (hybridized with fragment S3; Fig. 5) and mRNA (hybridized with fragment S4; result not shown). The size of the signal corresponded to the size of the full-length cDNA. Northern blot hybridizations with S3, S4 or further probes from other parts of SmDia did not provide evidence for alternatively spliced mRNA products (Fig. 5; results not shown).

**Figure 6:**
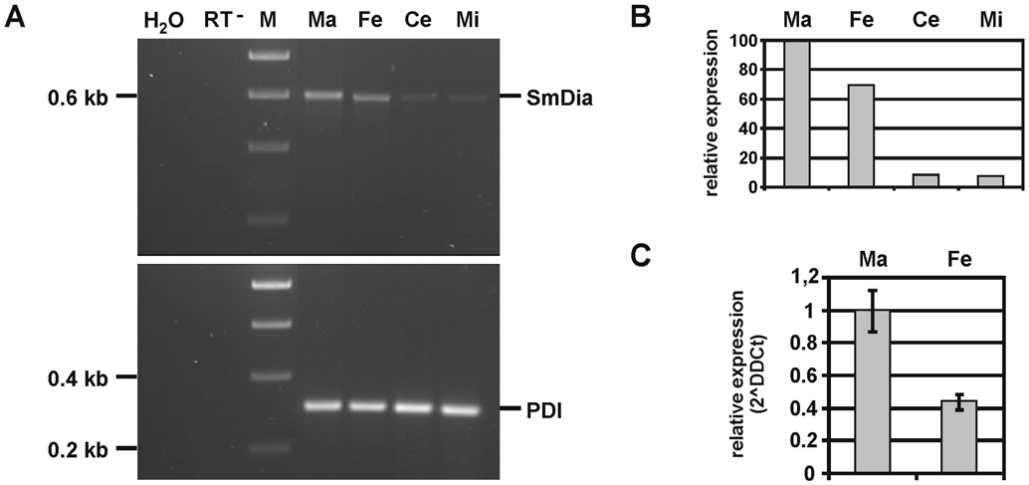
Gender and stage-specific expression of SmDia compared to PDI. Semi-quantitative RT-PCR analysis (A), densitometric quantification (B), and real-time PCR analysis (C). RT-PCR and qPCR experiments were performed with total RNA from adult worms (females or males) and free-living larval stages (miracidia and cercariae). A: Using SmDia gene-specific primers (SIV5′/SIV3′), RT-PCR products of the expected size of 0.6 kb were obtained from each template, males (Ma), females (Fe), cercariae (Ce), or miracidia (Mi). For control, RNA from mixed worm pools (mixed-sex adult worms and larvae) was used without initial cDNA synthesis (RT^−^). As an additional negative control, PCR was done without any template (H_2_O). In the reactions for quantification, primers against the constitutively transcribed protein disulfide-isomerase gene (PDI, 33) were used. B: Result of the densitometric quantification using the PDI RT-PCR products for normalization (n = 1). C: qPCR results, again taking PDI as reference (n = 3).

To analyze the occurrence of SmDia transcripts during schistosome development, an initial semi-quantitative RT-PCR experiment was performed with total RNA from adult worms and the free-living larval stages (miracidia and cercariae). Using gene-specific primers (SIV5′/SIV3′), RT-PCR products of the expected size (about 0.6 kb) were obtained from each template (Fig. 6A). The intensities of the amplification products were densitometrically quantified using the PDI RT-PCR amplification products for normalization. This first result indicated similar transcript levels of SmDia in miracidia and cercariae, but both were lower compared to adults (Fig. 6B). Between the adults, however, transcript levels differed with a bias towards males. To confirm this difference, subsequent qPCR analyses were performed comparing RNA from adult males and females. The result demonstrated that SmDia is most abundantly transcribed in males (Fig. 6C). The amount of SmDia transcripts detected by qPCR in females represented about 40% of that found in males.

### Detailed analyzes of the SmDia/SmTK3 interaction regions

**Figure 7:**
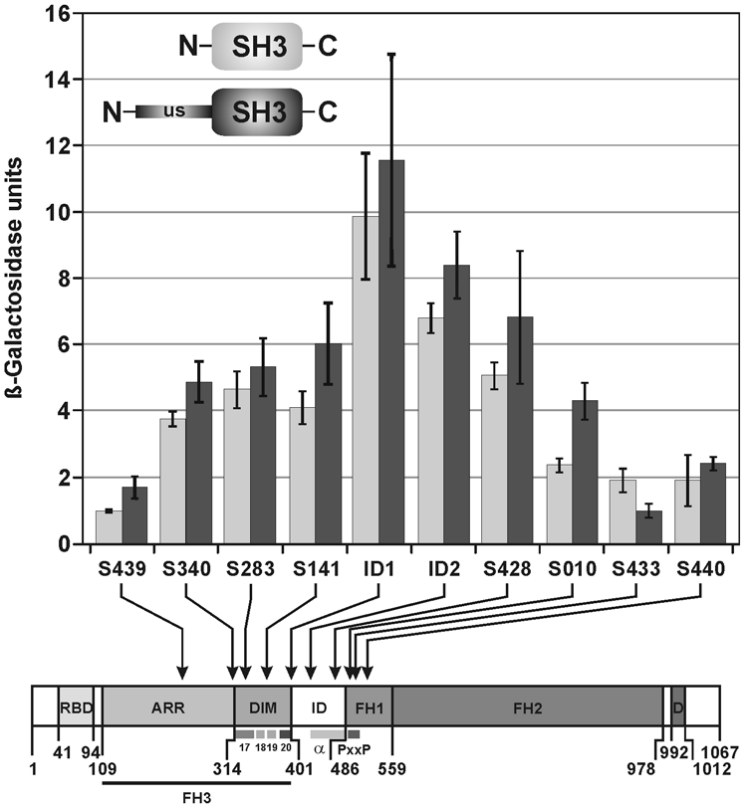
Contribution of the unique site on the binding of SmTK3 to different SmDia deletion constructs. A comparative β-Gal liquid assay showing the relative binding strength of 8 partial, N-terminally shortened SmDia clones of different length found in the Y2H library (S439-S440; see table 1), and two further deletion constructs generated by *in vitro* mutagenesis (ID1 and ID2) with the TK3-SH3- and TK3-US-SH3 bait-constructs of SmTK3 (n = 6). Indicated are the individual N-terminal end positions (arrows) of the different SmDia clones relative to the SmDia amino acid sequence (bottom; amino acid positions are given). Furthermore, the relative position of the PxxP core motif, the inter-domain region (ID) α-helix as predicted by computer analyses, as well as the 4 α-helices (17–20) within the DIM domain are specified. Light grey bars: interactions of the 10 SmDia clones with TK3-SH3; dark grey bars: interactions of the 10 SmDia clones with TK3-US-SH3.

Y2H screening of the *S. mansoni* cDNA library resulted in the identification of 8 SmDia clones with different lengths (see table 1, top) encoding fusion proteins, which are N-terminally shortened (see Fig. 4B). Since the binding characteristics of the unique site of Src kinases has not been closely defined yet [21], [27], [28], [41] we investigated the binding capacity of the 8 SmDia length variants in a comparative analysis with both SmTK3 baits. To this end, the 8 SmDia clones were individually re-transformed in yeast cells in combination with the bait constructs TK3-SH3 or TK3-US-SH3, respectively. In addition, by *in vitro* mutagenesis two different inter-domain constructs were generated (ID1, ID2). ID1 covered the complete inter-domain region, whereas ID2 comprised the C-terminal part of it representing one α-helix as predicted by computer analysis. As controls, the analogous domains of the novel Src kinase SmTK6 (Beckmann et al., in preparation), TK6-SH3 or the TK6-US-SH3 were used as baits. Semi-quantitative β-Gal liquid assays showed very weak binding of these SmTK6 constructs to SmDia, which indicated a non-specific binding (data not shown). Using the same assay, variable strengths of binding were observed for the SmTK3 constructs, depending on the extent of the N-terminal deletion (Fig. 7). For TK3-SH3 maximal binding was observed to SmDia clone ID1, which comprised amino acids 402–1067 including the complete inter-domain region, the FH1, FH2, and DAD domains. ID2 and the other shorter clone variants, as well as N-terminally elongated SmDia variants demonstrated a nearly gradual decrease in binding affinity following a Gaussian-like distribution. A similar pattern was detected by analyzing the results obtained with TK3-US-SH3 as bait. However, in nearly each case the strength of binding was enhanced when the unique site was present. This curve progression of binding efficiency of the individual clones pointed to decisive roles of the inter-domain region and the N-terminal part of the FH1 region for binding to the SH3 domain and the unique site, which is supported by analyzing the shorter clones (S010, S433, S440). They possess DAD, FH2, or parts of FH1 only, and nearly all show gradually decreasing binding strengths independent of the presence or absence of the SmTK3 unique site. A similar decrease in binding strengths was also observed for the longer SmDia clones (S141, S283, S340, S439), all of which contained the complete DIM region (S439, S340), or part of it (S238, S141). Since this region provides potential for dimerisation, it may competitively restrict the access of the SH3 domain/unique site to its binding region within SmDia. The negative binding effect was strongest when the C-terminal part of ARR was included (S439).

### SmDia and SmRho1 interact in the yeast two-hybrid system

Strong evidence has been obtained from cell culture studies that diaphanous proteins bridge Rho GTPases and Src kinases during signaling processes [21], [39]. Thus, we tested for a direct interaction of SmDia with SmRho1, a Rho GTPase homolog identified in *S. mansoni* [22]. Towards this end the Rho binding-domain (RBD domain) of SmDia, which serves as the docking region for the activated GTP-bound form of Rho [17], [20], was used as bait.

Rho GTPases as well as other small GTPases are membrane-associated intracellular proteins. They possess a CAAX-box motif at their C-terminal ends, which is prenylated to enable membrane anchoring [19]. In the Y2H system, however, membrane localization of a candidate protein could counteract the nuclear localization of the potential binding partners, thus preventing the initiation of reporter-gene transcription. To avoid this, we removed the CAAX box from the wildtype full-length SmRho1 sequence in the prey construct (ΔCAAX SmRho1) (see Materials and Methods). Since the C-terminal end of Rho GTPases has no catalytic function, being exclusively important for membrane localization [19], this deletion was expected not to influence the GDP/GTP-binding capacity of SmRho1, or its potential to interact with binding partners.

zFurthermore, an interaction of two potential binding partners in the used yeast system leads to the association of the DNA binding and activation domains thus forming a chimeric, functional Gal4 transcription factor. This requires a persistent binding of both partners. However, the activated GTP-bound state of Rho GTPases is subject to rapid turnover [19]. Therefore, due to its transient nature, GTP-bound wildtype SmRho1 may not be able to bind to the RBD of SmDia with an efficiency sufficient to induce reporter-gene activity. To overcome this hypothetical obstacle, we introduced point mutations into the ΔCAAX-SmRho1 sequence. Single point mutations at positions 15 (G15V) or 64 (Q64L) significantly elongated the half-time of the GTP-bound state of small GTPases by reducing intrinsic or GAP protein-mediated GTPase activity [19], [40]. Therefore, we assumed that the G15V or Q64L ΔCAAX-SmRho1 variants bind to SmDia-RBD with an affinity sufficient to induce reporter-gene activity.

**Figure 8:**
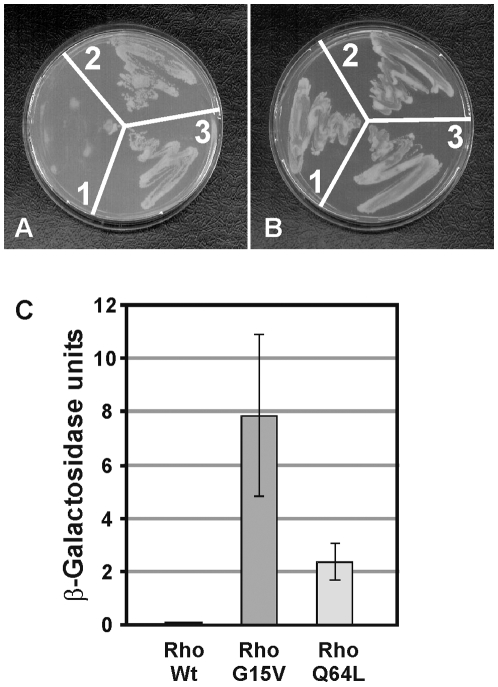
SmDia/SmRho1-interaction in yeast. Qualitative (A, B) and quantitative (C) Y2H analyses of SmDia-SmRho1 interaction. As bait, the RBD domain of SmDia was cloned into the bait vector pGBKT7 in fusion with the Gal4 DNA-binding domain (pGBKT7-DiaRBD). As prey, a mutated form of SmRho1 was generated lacking the CAAX-box at its C-terminal end and cloned into the pACT2 prey vector. Subsequently, this construct was employed to generate two variants containing point mutations at positions 15 and 64 (G15V or Q64L), respectively. A: Yeast cells were transformed with the pGBKT7-DiaRBD and the SmRho1 variants RhoWt (wildtype; 1), RhoG15V (2), or RhoQ64L (3) in pACT2. Transformed yeast cells were selected for the presence of the plasmids and for reporter gene expression (Trp^−^/Leu^−^/Ade^−^/His^−^). B: Control, the same transformed yeast cells grew on media selective for the presence of the plasmids (Trp^−^/Leu^−^). C: The β-Gal liquid assays (n = 2) demonstrated a reduced binding affinity for SmRho1 wildtype compared to the mutant forms RhoG15V or RhoQ64L, as indicated in A.

As shown in Figure 8 (A), yeast cells cotransformed with the SmDia-RBD construct and one of the point-mutated SmRho1 variants grew under conditions, which were selective for the presence of both plasmids and for reporter-gene activity (Trp^−^/Leu^−^/Ade^−^/His^−^). In contrast, yeast cells containing the wildtype (G15 and Q64) form of ΔCAAX-SmRho1 revealed a significantly reduced growth on this fourfold selection medium. A parallel selection of the same transformed yeast cells on twofold selection media (Trp^−^/Leu^−^) assured the presence of both plasmids (Fig. 8B). Since growth on fourfold selection media requires the interaction of the bait and prey proteins, we supposed that only the point-mutated ΔCAAX-SmRho1 variants were able to efficiently bind to SmDia-RBD. Quantitative analyses performing β-Gal liquid assays finally confirmed this assumption showing a reduced binding efficiency of the SmRho1 wildtype compared to the mutant forms (Fig. 8C). Additionally, it was found that the G15V variant bound with a higher affinity to SmDia-RBD than the Q64L variant. This indicated a higher stability of the G15V variant with respect to the maintenance of the GTP-bound state, a finding that corresponds to similar studies in other systems [40].

From these data we conclude that the GTPase SmRho1 directly interacts with SmDia.

### Localization of SmDia and SmRho1 transcripts

**Figure 9:**
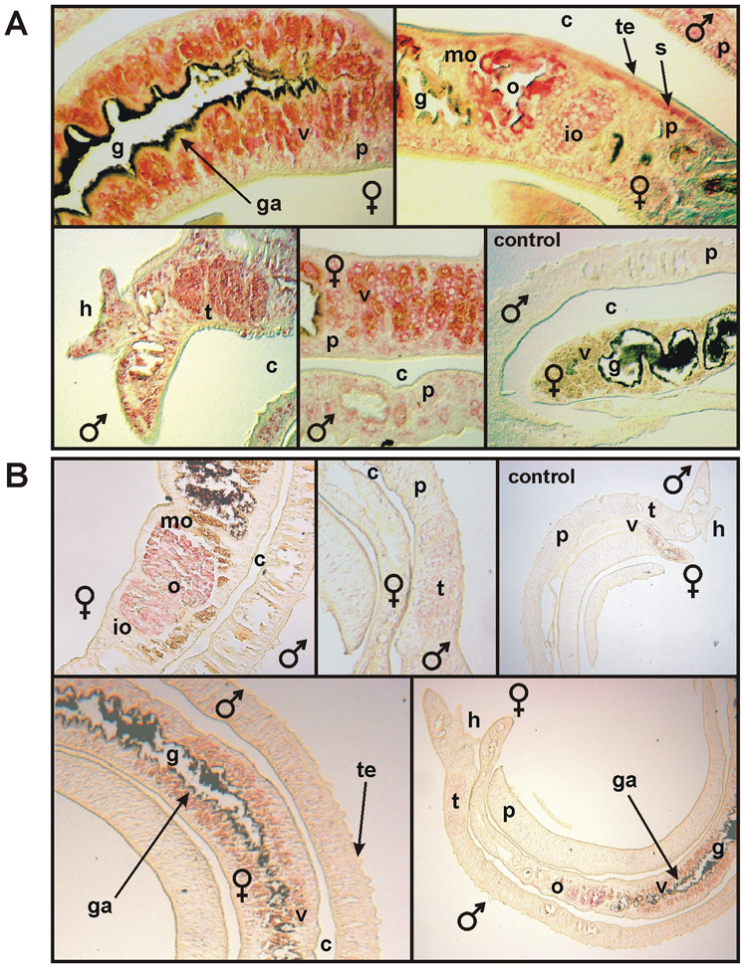
Tissue-specific transcription of SmDia and SmRho1 in adult *S. mansoni.* * In situ* hybridization experiments to localize SmDia (A) and SmRho1 (B) using DIG-labeled antisense- and sense-RNA probes of SmDia (fragment S4, see Fig. 3), or SmRho1 (nearly the complete cDNA, 564 bp). A: SmDia transcripts were detected in the cytoplasm of vitelline cells (v) and in the oocytes within the ovary (o) of the female, in the testes (t) of the male, and in the parenchyma (p) and the subtegumental (s) regions of both genders. Within the ovary signal intensity was stronger in mature oocytes (mo) compared to immature oocytes (io). No signal was found in the gastrodermis (ga) or in the tegument (te) of both genders. As negative control sense transcripts of SmDia (fragment S4) were used, which provided no signal. c, gynaecophoric canal; h, head sucker; g, gut. B: SmRho1 transcripts were also detected in the cytoplasm of vitelline cells and oocytes of the female, in the testes of the male, and in parenchyma and subtegumental regions of both genders. SmRho1 sense transcripts provided no signal (control).

*In situ* hybridization experiments were performed to investigate the tissue-specific distribution of SmDia and SmRho1 transcripts in adult schistosomes. Using a SmDia antisense probe (fragment S4, see Fig. 4A), signals were detected in the cytoplasm of vitelline cells and oocytes of the female, in the testes of the male, and in the parenchyma and the subtegumental area of both genders (Fig. 9A). Within the ovary, stronger signals were detected in its posterior part, which contains mature oocytes. No signals were found in the gastrodermis, or in the tegument of both genders. Gonad-specific and parenchymatic transcription of SmDia in adult *S. mansoni* correlated with *in situ* hybridization results previously obtained with SmTK3, which was shown also to be expressed in the vitellarium and the oocytes of the female, the testes of the male, and in the parenchyma of both genders [13].

Since we obtained evidence in this study for an interaction of SmDia and the GTPase SmRho1 of *S. mansoni* further *in situ* hybridization experiments were done. Using an antisense probe of SmRho1, signals were detected in the cytoplasm of vitelline cells and oocytes of the female, in the testes of the male, in the parenchyma, and in subtegumental regions of both genders (Fig. 9B). These data demonstrate the colocalization of the transcripts of SmRho1 and SmDia together with SmTK3 in the gonads and in the parenchyma of adult schistosomes.

## Discussion

Cellular PTKs function as transmitter molecules in signaling pathways regulating important biological processes such as cell proliferation or cytoskeletal rearrangement [27], [41]. In schistosomes, PTKs with structural and biochemical characteristics of Src kinases have been isolated and characterized such as SmTK3 [12], [13]. In transient transfection experiments with HEK293 cells, recombinant SmTK3 was able to phosphorylate the Src-kinase substrate p130Cas, an intracellular scaffolding protein involved in the organisation of the cytoskeleton [13]. In the same study, preliminary screenings of a *Drosophila* Y2H library with the unique site and the SH3 domain of SmTK3 as baits identified the Abl-interacting protein dAbi, vinculin, and tubulin as strongest binding partners. Since all these proteins fulfill diverse functions during cytoskeletal organization, and since signaling processes are functionally conserved in nature, it was hypothesized that SmTK3 might be involved in signal transduction cascades affecting cell architecture in *S. mansoni*, too. Furthermore, a tissue-specific role for SmTK3 was postulated since localization experiments identified expression products in the gonads of *S. mansoni* males or females. Finally, it was demonstrated that the Src kinase-specific inhibitor Herbimycin A caused a significant reduction of mitogenic activity and egg production in paired females *in vitro*, and negatively influenced SmTK3 stability [15].

Due to the accumulating evidence that SmTK3 is an important molecule for reproduction, we intended to identify its binding partners in schistosomes to discover signaling pathways in which this Src kinase may play a role. To this end, a Y2H library was established using poly(A^+^)-RNA of female and male *S. mansoni*. As bait for screening we used the SH3 domain in combination with the unique site (TK3-US-SH3). It had been shown before that SH3 domains bind to proline-rich sequences (PxxP) of partner molecules acting downstream in a signaling hierarchy [25], [42]. Furthermore, it was supposed that the specificity of SH3 binding can be enhanced by the unique site [27], [28]. Following extensive pre-selection strategies, 39 clones representing 9 different groups of molecules were obtained as potential interacting partners of SmTK3. According to sequencing and data-base analyses one group (I; Table 1) may represent pericentrin B-like genes, although homology was not significant. Pericentrins are centrosomal proteins involved in microtubule-organization [43]. Although interactions with SmTK3 baits was only of medium strength relative to others, the high number of group I-clones indicates that the appropriate gene may be abundantly transcribed. Another group (H) showed sequence homology to Smad proteins, which are involved in TGFβ signaling. Since a cross-talk between Src-kinase and TGFβ-signaling pathways has been hypothesized in schistosome reproductive organs [12], this molecule may be a candidate for mediating between these pathways. The SH3-domain binding-protein (group G) revealed homology to CR16 of *Rattus norvegicus*, which is a MAP kinase substrate [44]. Recent studies show that CR16 belongs to the verprolin family of proteins, which has actin-binding activity being involved in processes of cell growth, cytoskeleton organization, acting binding and endocytosis [45]. With respect to its amino acid sequence, verprolin has significant similarities to vinculin, which had been identified earlier with SmTK3-SH3 baits using the *Drosophila* Y2H-library [13]. Quinolinic acid phosphoribosyltransferase (Qprt; see group F) is the catabolic enzyme of quinolinic acid, an excitotoxic compound present in the mammalian CNS [46]. For Qprt as well as for guanine-7 methyl transferases (group E) it is not known whether they interact with Src kinases, although an involvement of methyl transferases in signaling processes has been shown [47]. YME-1 (group D) is a metalloprotease, homologs of which have been found in yeast, *C. elegans* and *Drosophila* [48]. Except for metalloproteases of the ADAM desintegrin family [49], an interaction of Src kinases and metalloproteases has not yet been described. Quantitative analyses revealed the BAF60 subunit of the SWI/SNF complex as the strongest binding partner of the used SmTK3 bait proteins (group C). Studies in eukaryotes have shown that the multi-subunit complex SWI/SNF exerts ATP-dependent chromatin-remodeling activities and is implicated in DNA-damage responses, transcriptional activation, sliding of nucleosomes, and alteration of histone-DNA contacts [50]–[52]. To our knowledge, no direct interaction between Src kinases and a BAF60 subunit, which fulfills its function within the nucleus, has been described yet. On the basis of the finding that BAF60 is phosphorylated by p38-kinase during skeletal myogenesis, however, it has been hypothesized that SWI/SNF subunits may be targets of signaling-dependent kinase cascades [53]. Recently, strong evidence was obtained that nuclear tyrosine phosphorylation by Src kinases plays an important role in chromatin structural changes upon growth factor stimulation [54]. Therefore, it is tempting to speculate that SmTK3 phosphorylates schistosome BAF60 within the nucleus, thereby influencing chromatin remodeling activities. The translation initiation factor eIF-4γ (group B) is part of the eIF-4 complex, which is involved in mRNA cap recognition, resolution of 5′secondary structures in mRNAs, ribosome guiding, and translational control [55]. Phosphorylation of subunits of the e-IF complex by MAP kinases is important for translation activation. Furthermore, recent data indicate a role of eIF4-family members in the control of translation of gene transcripts involved in cell growth, proliferation, and bioenergetics [55]. Although eIF-4γ has a number of potential tyrosine phosphorylation sites, to our knowledge there is no evidence in the literature yet for a role of Src kinases to phosphorylate these sites.

Diaphanous homologs finally represented the most frequently occurring clones within an allocable group (A). Sequence analyses showed that all 8 clones represented the same molecule in different lengths. SmDia is a single copy gene without introns encoding a transcript of 3.5 kb, which occurs most abundantly in males, and which is probably not alternatively spliced. The mRNA of SmDia codes for a protein of 1067 amino acids and reveals all hallmark domains typical for diaphanous-related formins. To investigate binding characteristics of the SH3 domain and the unique site, Y2H analyses were performed by retransformation experiments with truncated SmDia clones. Those lacking the complete N-terminus of the FH1 domain showed comparatively weak binding to both baits (S440, S433). Within the prolin-rich FH1 domain only the N-terminal PxxP motifs (between amino acid positions 491–501; see boxed amino acids in Fig. 4B) are putative candidates for contributing to SmTK3 binding because reporter-gene activity started to increase when this region was present (Fig. 4B and Fig. 7, compare reporter gene activities of clones S440, S433 and S010). However, the PxxP motifs are obviously not sufficient for maximal binding activity since part of the inter-domain region upstream enhanced binding activity (Fig. 7, S428). Enlarging the inter-domain portion enhanced binding successively (Fig. 7, S428, ID2, ID1) indicating that not only the α-helix within the C-terminal part but the complete inter-domain region contributed to binding efficiency. Interestingly, the FH1 N-terminal PxxP motifs seemed to bind stronger to the unique site-containing SH3 domain construct since the reporter gene activity determined for clones S433 and S010 exhibited a significant increase only for TK3-US-SH3, but not for TK-SH3. Since the values of reporter gene activity between TK3-US-SH3 or TK-SH3 with respect to the clones S010, S428, ID2, and ID1 showed a constant average difference it is tempting to speculate that the unique site of SmTK3 is mainly responsible for binding to the PxxP-motif region, whereas the SH3 domain of SmTK3 strongly reinforces the general binding efficiency by contacting the inter-domain region. As there are no classical PxxP motifs within the inter-domain region, we assume that it contains other not yet clearly definable, atypical binding motifs as alternative targets of the SmTK3-SH3 domain. In support of this assumption, recent studies showed that the binding specificity of SH3 domains is far more diverse than previously appreciated [26], [42]. SmDia/SmTK3 binding efficiency was negatively influenced when regions upstream of the inter-domain region of SmDia were part of the constructs. Since all of these contained parts of or the entire DIM region, it seems feasible that SmDia dimerization competed with the binding to the SmTK3 baits.

To obtain experimental evidence for the assumption that SmDia also in schistosomes bridges Rho GTPases and Src kinases in signaling processes, additional Y2H experiments were done that provided evidence for SmDia-SmRho1 interaction (Fig. 8). *In situ* hybridization experiments demonstrated the transcription of SmDia and SmRho1 in the ovary and vitellarium of the female, and the testes of the male (Fig. 9). Within the ovary, signal intensities of SmDia as well as SmRho1 increased with the size of the oocytes. Although *in situ* hybridization is not a quantitative method, these corresponding results may indicate that SmDia and SmRho1 transcription increase during oocyte maturation. The colocalization data in this and a previous study with SmTK3 [13] provide additional support for the conclusion that all three molecules interact in the gonads of schistosomes. A role of Dia proteins in reproductive organs has been detected before only in *Drosophila*. Here, these proteins function as regulators of mitotic and meiotic cytokinesis in both the germline and soma during oogenesis and spermatogenesis [reviewed in 56].

**Figure 10:**
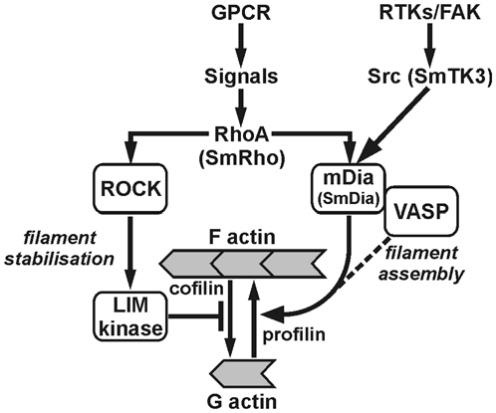
Model of signaling pathways involving Rho, Src, and Dia in cytoskeleton organization. Based on the existing model of Tominaga et al. (2000) and Grosse et al. (2003) (21, 39), we suggest the existence of a minimum of two conserved cooperative signal-transduction pathways in *S. mansoni* involving the small GTPase SmRho1 and the Src kinase SmTK3 that bridge at SmDia to organize cytoskeletal events in the reproductive organs of males and females (figure modified from 39).

Based on our results and on previous data from cell culture studies [21], [39], [57], we suggest the existence of a minimum of two conserved cooperative signal-transduction pathways, involving Rho and Src, that bridge at Dia to organize cytoskeletal events in the gonads of *S. mansoni* males and females (Fig. 10). These pathways are initiated by receptor tyrosine-kinases and G-protein coupled receptors, of which many candidates exist in the schistosome genome. Besides their role in cytoskeletal organization Rho, Src, and Dia may also be associated with endosomal mobility via actin filaments [18], [58], [59].

With respect to the actual knowledge [17], [18], [21], [56], [59] this study is not only the first evidential report on the existence of these cooperating pathways in the reproductive organs of a parasite, but also in gonads of eukaryotes in general. The proposed model (Fig. 10) helps to interpret the decrease of mitogenic activity, egg production and SmTK3 stability of paired females in response to Herbimycin A inhibitor-treatment [15] in more detail. Since Src kinases play a role in actin assembly, the decrease in mitogenic activity and egg production may have resulted from a reduced capacity of cytoskeletal organization and/or endosomal transport activities in the gonads, especially within the mitotically highly active vitellarium and the ovary. The fact that the schistosome tyrosine kinases identified yet are expressed throughout development fulfilling roles not only in reproduction but probably also in other growth and developmental processes strengthen the concept of specific tyrosine-kinase inhibitors as suitable candidates for novel chemotherapeutic strategies to fight schistosomes [12], [60]–[62].
